# The roles of mesenchymal stem cells in tumor inflammatory microenvironment

**DOI:** 10.1186/1756-8722-7-14

**Published:** 2014-02-06

**Authors:** Zhao Sun, Shihua Wang, Robert Chunhua Zhao

**Affiliations:** 1 Department of Oncology, Peking Union Medical College Hospital, Chinese Academy of Medical Sciences and Peking Union Medical College, Beijing, People’s Republic of China; 2 Center of Excellence in Tissue Engineering, Institute of Basic Medical Sciences and School of Basic Medicine, Chinese Academy of Medical Sciences and Peking Union Medical College, Beijing, People’s Republic of China; 3 Center of Translational medicine Peking Union Medical College Hospital, Chinese Academy of Medical Sciences and Peking Union Medical College, Beijing, People’s Republic of China

**Keywords:** Mesenchymal stem cell, Tumor, Inflammatory microenvironment

## Abstract

Tumor behavior is not entirely determined by tumor cells. Studies have demonstrated that a variety of non-tumor cells in the tumor microenvironment affect tumor behavior; thus, a new focus of cancer research has been the development of novel cancer treatment ideas and therapeutic targets based on the effects of these cells. Mesenchymal stem cells (MSCs) are an important component of the tumor microenvironment; however, previous studies have produced controversial results regarding whether MSCs promote or inhibit tumor growth and progression. In particular, Naïve MSCs and tumor-derived MSCs (T-MSCs) have different functions. Naïve MSCs could exert bidirectional effects on tumors because these cells can both promote and inhibit tumor progression while T-MSCs promote tumor progression due to influences from the tumor itself and from the inflammatory tumor microenvironment. As an unhealed wound, tumor produces a continuous source of inflammatory mediators and causes aggregation of numerous inflammatory cells, which constitute an inflammatory microenvironment. Inflammatory factors can induce homing of circulating MSCs and MSCs in adjacent tissues into tumors, which are then being “educated” by the tumor microenvironment to support tumor growth. T-MSCs could recruit more immune cells into the tumor microenvironment, increase the proportion of cancer stem cells and promote tumor angiogenesis, further supporting tumor progression. However, as *plasti*city is a fundamental feature of MSCs, MSCs can also inhibit tumors by activating various MSC-based signaling pathways. Studies of the mechanisms by which interactions among tumors, MSCs, and the inflammatory microenvironment occur and methods to disrupt these interactions will likely reveal new targets for cancer therapy.

## Introduction

In 2000, Hanahan and Weinberg summarized the hallmarks of cancer, which included the following capabilities: self-sufficiency with regard to growth signals, apoptosis evasion, insensitivity to anti-growth signals, sustained angiogenesis, tissue invasion & metastasis, and a limitless replicative potential [1]. However, recent studies have demonstrated that tumor behavior is not completely determined by tumor cells alone; in particular, the roles of cytokines and non-tumor cells in the tumor microenvironment during tumorigenesis and tumor development have drawn increased research attention [2]. Tumors are now regarded as chronic injuries that are difficult to heal [3], and inflammation is known to play an important role in tumorigenesis, tumor progression, and metastasis [4,5].

The main cytokines present in the inflammatory tumor microenvironment include inflammatory cytokines such as tumor necrosis factor α (TNF-α) [6], interferon-gamma (IFN-γ) [7], interleukin-6 (IL-6) [8,9], IL-8 [8], IL-1, and transforming growth factor-beta (TGF-β); growth factors such as hepatocyte growth factor (HGF), platelet-derived growth factor (PDGF), and vascular endothelial growth factor (VEGF); chemokines such as stromal cell-derived factor-1 (SDF-1) [10]; and other factors such as matrix metalloproteinases. The main non-tumor cells present in the inflammatory tumor microenvironment include inflammatory cells such as lymphocytes, macrophages, and myeloid-derived suppressor cells [11]; vascular endothelial cells; and tumor-associated stromal cells such as tumor-associated fibroblasts (TAFs) and MSCs [12]. These non-tumor cells support and facilitate tumor development.

MSCs were first discovered in the bone marrow, although they account for only 1/10^5^ nucleated bone marrow cells. MSCs and MSC-differentiated stromal cells play supportive roles in hematopoiesis and hold potential for a variety of diseases [13]. Chromosomal or functional abnormalities in MSCs could be present in various malignant hematological diseases such as myelodysplastic syndromes [14,15], lymphocytic leukemia [16], multiple myeloma [17], acute myeloid leukemia [18,19], and chronic myeloid leukemia [20]. Defects in MSCs can lead to hematopoietic abnormalities. It has been conjectured that MSCs might also exhibit functional abnormalities in solid tumors. MSCs are not particularly prevalent in solid tumor tissues, accounting for only 0.01% of the total cell number within these tissues [21]. However, the role of MSCs in this context has drawn increasing attention from researchers.

Prior reports have produced controversial results regarding the roles of MSCs in solid tumors. Certain studies have demonstrated that MSCs can inhibit tumor proliferation [22] and promote tumor cell apoptosis [23], whereas other investigations have revealed that MSCs promote the epithelial-mesenchymal transition (EMT) during tumor progression [24] and increase the proportion of stem cells in tumors [25]. MSC membranes express receptors for a number of different growth factors and inflammatory cytokines, and MSCs exhibit plasticity; specifically, in different microenvironments or under different induction conditions, MSCs can differentiate into cells of various types or functions that play different roles in tumor development. Therefore, studies of the tumor-specific roles of MSCs must account for the effects of the inflammatory tumor microenvironment.

Below, we will separately discuss the effects of tumors and the tumor inflammatory microenvironment on MSC homing and differentiation, the different functions of naïve MSCs from normal tissues and tumor tissue-derived MSCs (tumor tissue-educated MSCs; T-MSCs), and the potential mechanisms by which tumors “educate” MSCs.

### The mechanisms of MSC homing to tumor tissues

The influence of the inflammatory tumor microenvironment enables MSCs to specifically home to tumor tissues while avoiding peritumoral normal tissues [24]. MSCs can home to nearly all inflammation sites, including ischemic myocardial tissues, wounded skin regions, and the gastrointestinal mucosa after radiotherapy.

Researchers have established a tumor model in which MSCs isolated from fluorescent mice are introduced into non-fluorescent mice (by either replacing the bone marrow cells of the tumor-bearing mice with enhanced green fluorescent protein (EGFP)-positive bone marrow cells or by subcutaneously transplanting EGFP-positive adipose tissues into tumor-bearing mice) [26]. The results from this model have demonstrated that MSCs in mouse tumor tissues might be derived from the bone marrow; in other words, these MSCs might be circulating bone marrow MSCs. Adipose tissues near tumors provide another source of MSCs in tumors. There are certain differences in the outcomes produced in tumor tissues by MSCs derived from these two sources.

**Figure 1:**
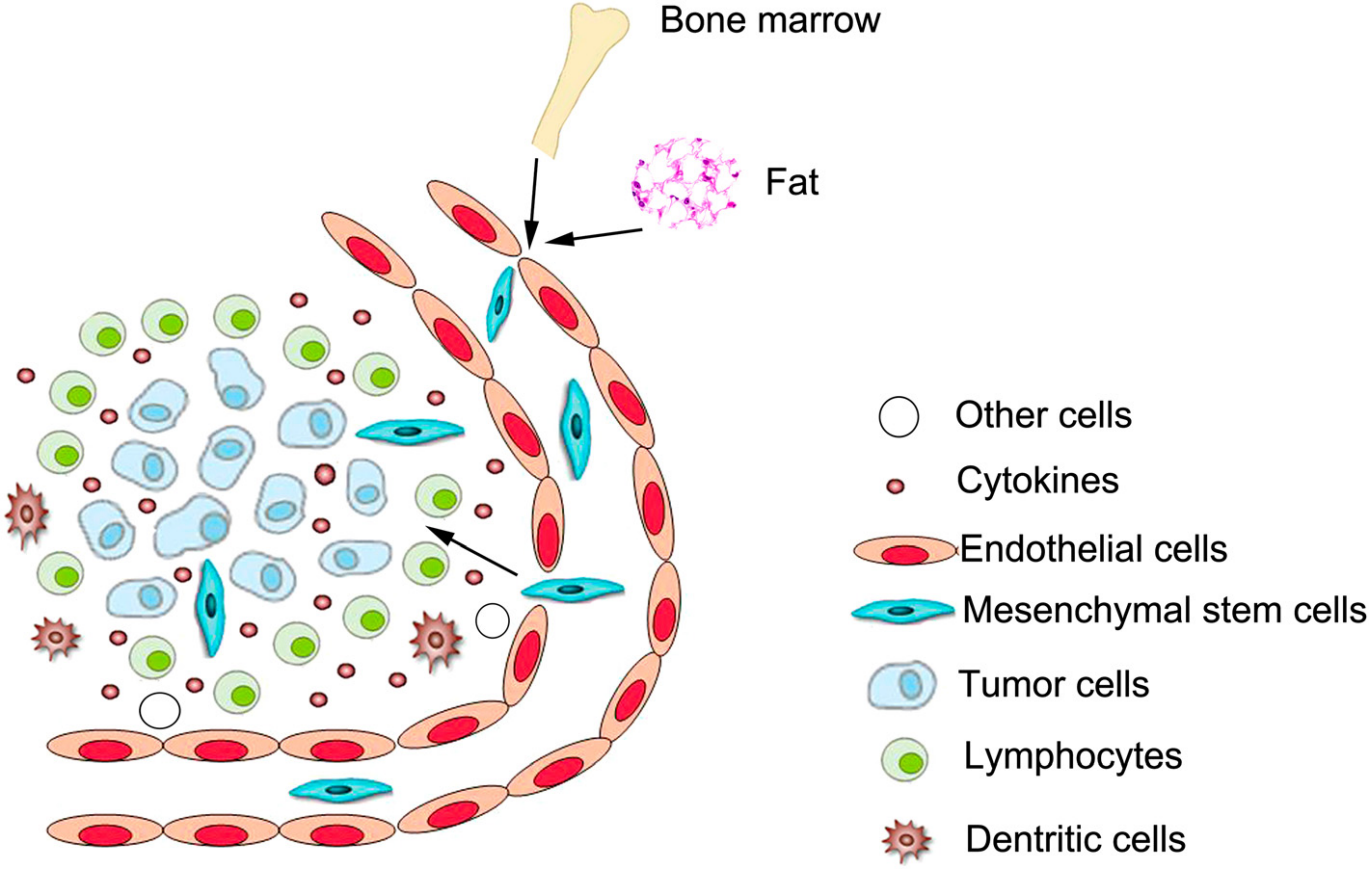
**Homing of MSCs to tumors.** As a wound that doesn’t heal, tumor produces a continuous source of inflammatory mediators and causes aggregation of numerous inflammatory cells, which constitute an inflammatory microenvironment. Inflammatory mediators could attract MSCs from bone marrow or adjacent adipose tissues and these MSCs become a major component of tumor microenvironment.

Tumors and their microenvironments induce MSC homing through mechanisms that depend mainly on various inflammatory cytokines, chemokines, growth factors, and other factors (Figure 1). These major factors in MSC homing are discussed below. First, inflammatory cytokines. The first step in the homing of circulating MSCs to tumor tissue is MSCs adhesion to the vascular endothelium, followed by crossing of the endothelial layer. Uchibori et al. and Teo et al. detected high concentrations of TNF-α in tumor tissues. TNF-α upregulates vascular cell adhesion molecule-1 (VCAM-1) expression on MSCs, thereby promoting the adhesion of MSCs to endothelial cells. IL-1β and IFN-γ can produce similar effects [27,28]. Because MSCs express IL-6 receptors, high IL-6 concentrations in tumor tissues could directly induce the accumulation of MSCs in these tissues. Additionally, Liu et al. found that IL-6 induces high expression levels of the CXC motif ligand (CXCL) chemokines CXCL7, CXCL6, and CXCL5 in MSCs [29]. Second, chemokines. Tumor cells secrete large quantities of SDF-1 [30,31], and MSCs express the SDF-1 receptor CXC chemokine receptor 4 (CXCR4). Therefore, an SDF-1 concentration gradient could induce MSC migration [32]. Third, growth factors. PDGF, HGF, and other growth factors could induce the migration of MSCs to tumor cells [27,33]. Finally, other factors. Various other factors such as hypoxia-inducible factor 1 (HIF-1) also play crucial roles in MSC homing. Tumors typically grow under hypoxic conditions. In hypoxic environments, breast cancer cells express high levels of HIF-1, which promotes the expression of CXC chemokine receptor 3 (CXCR3) and CXC chemokine receptor 5 (CXCR5) on breast cancer cell lines. MSCs express high levels of CXCL10 and the chemokine CC motif ligand 5 (CCL5). Therefore, MSCs are recruited to breast cancer areas and can promote breast cancer metastasis to the lung and lymph nodes. Thus, the homing of MSCs to tumors results from the combined effects of multiple cytokines. The major cytokines involved in this process are summarized in Table 1 (below).

**Table 1:** Cytokines involved in the homing of MSCs to tumors

**Category**	**Literature references**
*Inflammatory cytokines*	
*TNF-α*	[27,28]
*IFN-γ*	[27]
*IL-1β*	[27]
*IL-6*	[29,34]
*IL-8*	[35]
*Growth factors*	
*TGF-β*	[36]
*PGF*	[33]
*PDGF*	[27]
*HGF*	[27]
*Chemokines*	
*SDF-1/CXCR4*	[30,31]
*MMP*	[37]
*VCAM-1*	[38]
*CXCL (GRO-a)*	[29,35]
*MCP-1*	[39]
*Other factors*	
*HIF-1*	[33]
*LL-37*	[40]

### The outcomes of MSC homing to tumor tissues

MSCs are known to exhibit plasticity. MSCs that home to tumors can exist in 3 forms due to the effects of the inflammatory tumor microenvironment.

MSCs that are recruited to tumor tissues might continue to exist as MSCs. Numerous studies have confirmed that MSCs can be isolated from solid tumor tissues. These isolated MSCs are similar to normal tissue-derived MSCs (N-MSCs) with respect to morphology, phenotype, and multilineage differentiation capacity. However, T-MSCs and N-MSCs exhibit significant functional differences. We will describe these differences in detail later in this paper.

In tumor tissues, MSCs can also differentiate into tumor-associated myofibroblasts (TAFs; also known as cancer-associated myofibroblasts). TAFs and MSCs exhibit similar phenotypes; additionally, both cell types secrete similar cytokines. However, compared to MSCs, TAFs secrete significantly higher levels of TGF-β, VEGF, IL-4, and IL-10 [41,42]; moreover, TAFs express various specific markers such as α-smooth muscle actin (α-SMA), PDGF receptor-β (PDGFR-β), desmin, fibroblast-specific protein (FSP), and fibroblast activation protein (FAP) [26,43-46]. TAFs isolated from tumors or tumor-conditioned medium-induced myofibroblasts promote tumor growth, whereas myofibroblast-like cells derived from 5-azacytidine-induced MSCs do not produce this effect.

Tumor tissues contain large quantities of various angiogenic factors such as VEGF. *In vitro* studies have revealed that VEGF induction can induce MSCs to differentiate into vascular endothelial cells and even form three-dimensional vascular structures. However, the question of whether MSCs within tumors can differentiate into vascular endothelial cells *in vivo* remains controversial. In mouse models of breast cancer [44] and colon cancer [45], MSCs promoted tumor angiogenesis but did not differentiate into cluster of differentiation 31 (CD31) or von Willebrand factor (vWF)-positive endothelial cells. MSCs have only been known to differentiate into CD31-positive vascular endothelial cells, rather than into TAFs (α-SMA-positive cells), in a mouse model of melanoma [47]. An *in vivo* study by Kidd et al. found that the FAP^+^ and FSP^+^ TAF cells in tumor tissues were mainly derived from bone marrow MSCs, whereas vascular endothelial cells in tumor tissues were largely derived from nearby adipose tissues [26]. Thus, after circulating bone marrow MSCs and peritumoral adipose tissue-derived MSCs home to tumors, these two types of MSCs might differentiate along different pathways.

### The characteristics of T-MSCs

MSCs in tumor tissues are significantly affected by both tumor cells and the chronically inflammatory tumor microenvironment. In the following paragraphs, we will discuss the differences between T-MSCs and N-MSCs and the mechanisms that underlie these differences.

Many researchers have demonstrated that MSCs are non-neoplastic and chromosomally normal in solid tumors [48,49]. However, Lin et al. found that in certain colon cancer patients, MSCs isolated from tumor tissues exhibited the same chromosomal abnormalities that were present in the colon cancer cells, while in the remaining patients, the tumor cells exhibited chromosomal abnormalities but the MSCs remained chromosomally normal [50]. Moreover, p53 expression was observed to be generally low or absent in colon cancer-derived MSCs [50]. Wang et al. found that in MSCs, long-term stimulation with TNF-α, IFN-γ, and other factors could upregulate the expression of various proto-oncogenes such as c-Fos and c-Myc by activating the nuclear factor-kappa B (NF-kB) signaling pathway. T-MSCs and N-MSCs have similar phenotypes [48-52]; in particular, both types of MSCs express high levels of CD29, CD44, CD90, and CD105, but low levels of hematopoietic cell markers. The following differences between T-MSCs and N-MSCs have been observed: first, there are more MSCs in tumor tissues than in normal tissues. For instance, this phenomenon is quite evident in an examination of osteosarcoma tissues, as an average of 1117 clones of MSCs can be obtained from 10 [5] osteosarcoma cells, but only 1.3 clones can be obtained from 10 [5] normal bone marrow cells [49]. Examinations of specimens from cancer patients have also demonstrated that MSCs are more prevalent in tumor tissues than in adjacent normal tissues [53]. Second, T-MSCs exhibit a significantly greater proliferative capacity than N-MSCs. In particular, T-MSCs isolated from ovarian [48], pulmonary [46], stomach [54], and prostate [55] cancers, as well as pediatric neuroblastoma, teratoma, Ewing sarcoma, and rhabdomyosarcoma specimens [56], had shorter doubling times than N-MSCs. This phenomenon might occur because T-MSCs express a number of proliferation-related genes such as murine double minute 2, p21, and the zinc finger transcriptional factor sal-like protein 4 that are not expressed by N-MSCs [54]. Third, T-MSCs exhibit varying differentiation capabilities. Only a small subset of the MSCs isolated from pediatric neuroblastoma, teratoma, Ewing sarcoma, and rhabdomyosarcoma specimens could be induced to differentiate into adipocytes; however, these T-MSCs responded to osteogenic induction similarly as N-MSCs [56]. Fourth, T-MSCs exhibit stronger migratory capabilities than N-MSCs. Gastric cancer-derived MSCs were shown to possess particularly strong migratory capabilities [54]. Finally, T-MSCs exhibit strong drug resistance. MSCs derived from non-small-cell lung cancer were more resistant to cisplatin when compared with MSCs derived from normal lung tissue. Additional functional differences between T-MSCs and N-MSCs will be discussed further in the following section *that describes* the effects of T-MSCs on tumors.

### The effects of MSCs on tumors and the mechanisms that underlie these effects

MSCs might exhibit extremely different functions in distinct microenvironments because various signaling pathways can be activated in these cells. As a result, highly controversial results have been reported in prior studies that addressed the role of MSCs in tumor development. Therefore, it is very important to examine how MSCs in different states affect tumors. Generally, the MSCs used for clinical treatment are naïve MSCs that were cultured *in vitro* from normal tissue sources. These exogenous N-MSCs can carry exogenous genes, because they home to tumor tissues and are therefore expected to produce anti-tumor effects. Interactions between MSCs and tumor cells in tumor tissues could become a new cancer therapy target. In the following paragraphs, we will discuss the effects of naïve MSCs, T-MSCs, and MSCs that have been treated with various cytokines on tumors.

#### The effects of naïve (innate) MSCs on tumors

The earliest studies of MSCs and tumors focused on the effects of naïve MSCs on tumors. Naïve MSCs can inhibit tumor cell proliferation when co-cultured with tumor cells *in vitro*. Ramasamy et al. reported that N-MSCs could inhibit the proliferation of leukemia cell lines and solid tumor cell lines *in vitro*. This inhibitory effect of N-MSCs was dose-dependent, with stronger levels of inhibition observed at higher proportions of N-MSCs [57]. The underlying mechanism of this effect could involve the N-MSC-mediated secretion of soluble factors such as Dickkopf-related protein 1, which inhibits Wnt signaling pathways in tumor cells; Wnt pathway inhibition could, in turn, decrease c-Myc and Cyclin D2 expression and upregulate P21^CIP1^ and P27^KIP1^ expression, resulting in the suppression of cell cycle progression [22,58-60]. Naïve MSCs also induce apoptosis in tumor cells [23]. The mechanisms that underlie this effect could involve the upregulation of caspase 3, an apoptosis-related protease [58]. Additionally, naïve MSCs indirectly suppress tumor growth by inhibiting angiogenesis. N-MSCs can inhibit angiogenesis by inducing apoptosis in vascular endothelial cells [61,62] or by directly inhibiting vascular network formation [63]. However, opposing results have also been reported. In an animal model of prostate cancer, MSCs were shown to promote fibroblast growth factor 2 (FGF2) secretion by PC3 cells. MSCs were also observed to enhance the expression of endothelin-1 in colon cancer cell lines [64], thereby promoting tumor angiogenesis [30]. In a melanoma model, MSCs directly differentiated into vascular endothelial cells [47]. *Cancer stem cells (CSCs) contribute to tumorigenesis, metastasis and recurrence of tumors*[65]. Naïve MSCs can also enhance the proportion of CSCs in tumor cell populations. Nishimura et al. observed that co-culturing gastric cancer cell lines with MSCs increased the proportion of CD133-positive gastric cancer cells [25,65]. Similarly, Liu et al. found that co-culturing breast cancer cell lines and MSCs increased the percentage of Aldefluor + cells among the cancer cells. In animal models, MSCs were found to form stem cell niches in breast cancer tissues, with higher proportions of breast cancer stem cells present in the vicinities of these niches than at other sites [29]. Furthermore, naïve MSCs promote tumor cell migration. This effect might occur in response to various chemokines that are expressed by MSCs, including CCL5 [66,67], CXCR4 [68], intercellular adhesion molecules (ICAMs), and vascular cell adhesion molecules (VCAMs) [69]. These chemokines can function in a paracrine manner to not only promote tumor invasion and tumor migration but also facilitate EMT among tumor cells [70,71]. Finally, *in vivo*, naïve MSCs can suppress immune responses, thereby promoting tumor growth [72].

**Figure 2:**
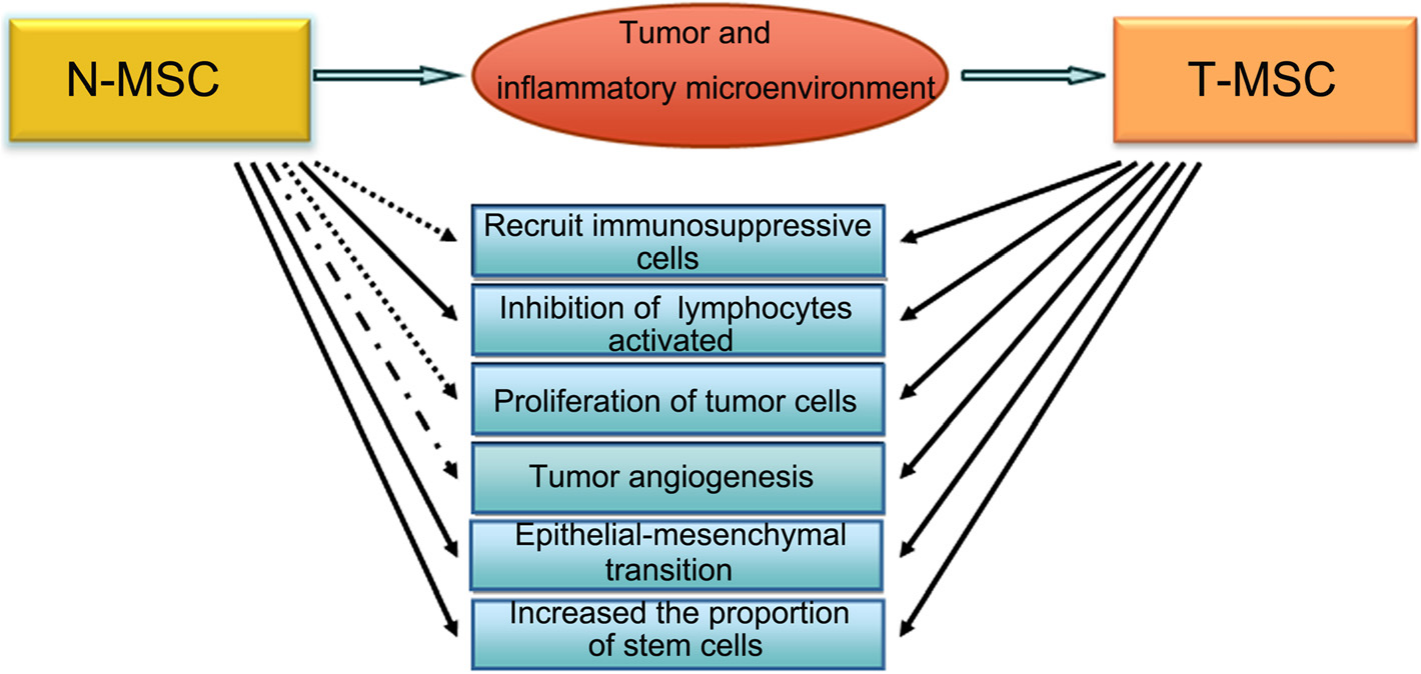
**Different effects of Naïve MSC (N-MSC) and (Tumor derived MSC (T- MSC) on tumors.** Naïve MSCs are ‘educated ‘by the tumor inflammatory microenvironment after homing to tumor tissues and transformation to T-MSCs exert different effects on tumor development.  Promote  Inhibit  Controversial.

In summary, naïve MSCs have been reported to exert bidirectional effects on tumor progression, *and different researchers have discrepancies on whether these MSCs exert tumor-promoting or tumor-suppressing effects.* These discrepancies might reflect differences in experimental conditions, as the results of each experiment reveal only one aspect of the mechanisms by which N-MSCs affect cancer cells. Additionally, the same researchers could obtain completely opposite results from *in vivo* and *in vitro* experiments. For instance, Ramasamy et al. reported that N-MSCs inhibited tumor proliferation *in vitro*, but that injecting MSCs could promote tumor growth in nude mice. Ramasamy et al. explained these findings by proposing that MSCs might form matrices to provide nutritional support or form cancer stem cell niches [57]. In fact, after *in vitro*-cultured N-MSCs home to tumors within animals, the N-MSCs will be clearly affected by these tumors, and the states and functions of the N-MSCs will be altered. Therefore, it is unsurprising that *in vivo* and *in vitro* studies of N-MSCs would yield differing results (Figure 2).

#### The effects of T-MSCs on tumors

##### T-MSCs exhibit stronger immunosuppressive activity than N-MSCs

Ren et al. found that, relative to bone marrow-derived MSCs (BM-MSCs), MSCs isolated from mouse lymphomas (L-MSCs) more strongly promoted tumor growth. This effect was mainly due to the high expression of CC motif chemokine receptor 2 (CCR2) ligands in these cells, which could recruit immunosuppressive cells such as CD11b^+^ Ly6C^+^ monocytes, F4/80^+^ macrophages, and CD11b^+^Ly6G^+^ neutrophils to lymphoid tissues. Monocyte/macrophage depletion or CCR2 gene knockout eliminated the L-MSC-mediated tumor-promoting effects [21]. Compared to MSCs isolated from normal breast tissues, MSCs derived from breast cancer tissues express higher levels of the immunosuppressive factors IL-4, IL-10, and TGF-β1. Furthermore, co-culturing peripheral blood mononuclear cells with breast cancer-derived MSCs increased the proportion of CD4^+^CD25^hi^Foxp3^+^ regulatory T cells [73]. Cervical cancer-derived MSCs significantly reduced the expression of human leukocyte antigen (HLA) class I molecules (HLA-A*0201) on the surface of cervical cancer line cells, thereby inhibiting the cytotoxic effects of antigen-specific T cells on these cervical cancer cells [74]. MSCs isolated from pediatric sarcomas inhibited the cytotoxic effects of natural killer (NK) cells by reducing the expression of the NK cell receptors NKp44 and NKp46 [56].

##### T-MSCs play a role in promoting EMT

Examinations of tissue sections from pancreatic cancer patients revealed that pancreatic cancer cells near T-MSCs exhibit reduced E-cadherin expression and elevated vimentin expression [75]. Additionally, CCL5 expression is upregulated in MSCs from prostate cancer and breast cancer cell lines. The upregulation of CCL5 expression is known to increase the expression of various EMT-related genes such as zinc finger E-box-binding homeobox 1, Snail, and CXCR4 [24,67].

##### T-MSCs promote tumor cell proliferation

McLean et al. reported that human ovarian cancer-derived MSCs promoted tumor cell proliferation in mice, with an accompanying increase in the proportion of Ki-67-positive tumor cells; in contrast, as noted above, N-MSCs did not significantly promote tumor proliferation [48].

##### T-MSCs increase the proportion of cancer stem cells

*In vitro* co-culture experiments have demonstrated that co-culture with T-MSCs can increase the proportion of cancer stem cells in ovarian cancer cell population. The mechanism that underlies this effect might be related to the greater expression levels of the bone morphogenetic proteins (BMPs) BMP2, BMP4, and BMP6 in ovarian cancer-derived MSCs, relative to N-MSCs. In particular, *in vitro* experiments indicated that BMP2 could simulate the effects of T-MSCs on cancer stem cells. Inhibition of the BMP signaling pathway abolished the MSC-mediated promotion of tumor growth [48]. Co-cultures of prostate cancer cells and BM-MSCs significantly increased the secretion of CCL5 by BM-MSCs. In prostate cancer cell lines, CCL5 enhanced the proportion of stem cells (CD133^+^ cells) in cell populations and promoted sphere formation [24].

To summarize the above-described findings, in contrast to naïve MSCs, T-MSCs indisputably promote tumor progression (Figure 2).

#### TLR4-mediated MSC activation

Tumors, the inflammatory tumor microenvironment, and T-MSCs typically form a mutually reinforcing vicious cycle. However, the activation of various surface receptors on MSCs could induce MSCs to adopt tumor-inhibiting phenotypes. Waterman et al. found that after activating Toll-like receptor 4 (TLR4) on MSCs with lipopolysaccharide (LPS) stimulation, MSCs secreted large quantities of various proinflammatory mediators, including IL-17, IL-3, monokine induced by gamma-interferon (MIG), macrophage inflammatory protein-1 beta (MIP1b), and granulocyte-macrophage colony-stimulating factor (GM-CSF). *In vitro* experiments have indicated that MSC^TLR4^, a proinflammatory MSC phenotype induced by TLR4 priming, supports T cell activation. MSC^TLR4^ promoted inflammation in a mouse model of inflammatory lung injury. In an ovarian cancer model, MSC^TLR4^ acted to recruit F4/80^+^ leukocytes (likely macrophages) and monocytes, whereas naïve MSCs recruited tumor-associated granulocytes [76]. Therefore, in a mouse model of ovarian cancer, MSC^TLR4^ did not promote tumor growth and metastasis. These results indicate that MSCs could be converted to a phenotype that is detrimental to tumor progression. Further studies are warranted to determine the effects of MSC^TLR4^ on EMT, stem cell proportions, and angiogenesis in the context of tumors.

### The mechanisms that produce differences between N-MSCs and T-MSCs

In the above discussions, we have described the differences between the effects of naïve MSCs and T-MSCs on tumors. These differences are mainly caused by the cytokines present in the inflammatory tumor microenvironment. MSCs express numerous cytokine receptors and therefore have the capacity to respond to various types of signals. Changes in the biological functions of MSCs can occur through ligand-receptor binding and the resulting activation of the corresponding signal pathways.

#### Inflammatory cytokine stimulation can enhance the immunosuppressive effects of MSCs

IFN-γ or TNF-α can enhance the immunosuppressive effects of MSCs [77,78]. Ren et al. found that, similarly to MSCs isolated from lymphomas, TNF-α-treated MSCs expressed high levels of CCR2 ligands and could recruit immunosuppressive cells [21]. In a mouse model of melanoma, the co-transplantation of B16 cells with IFN-γ and TNF-α-pretreated MSCs promoted tumor growth. IFN- γ and TNF-α pretreatment causes MSCs to secrete large quantities of inducible nitric oxide synthase (iNOS). This disrupted iNOS expression reverses the observed tumor-promoting effects of MSCs [79]. IL-1α pretreatment produces increased TGF-β expression in MSCs. The co-injection of prostate cancer cell lines with IL-1α-treated MSCs promotes tumor growth in mice. This tumor growth-promoting effect of IL-1α-pretreated MSCs can be blocked by siRNAs against TGF-β [80].

#### Inflammatory cytokines can enhance the tumor metastasis-promoting effects of MSCs

Inflammatory cytokine stimulation enhances the tumor metastasis-promoting effects of MSCs. There are several potential mechanisms that might underlie this effect; for example, inflammatory cytokines could enhance the ability of MSCs to promote EMT or upregulate the expression of chemokines or ligands by MSCs. MSC pretreatment with IFN-γ and TNF-α upregulates TGF-β expression in the treated MSCs, and TGF-β can promote EMT. Experiments both *in vitro* and in animal models of liver cancer have demonstrated that, compared with conditioned medium from untreated and single-factor treatment groups, conditioned medium from MSCs pretreated with IFN-γ and TNF-α significantly promoted invasion by liver cancer cells. In mouse models of liver cancer, conditioned medium from pretreated MSCs significantly increased metastasis [81]. MSCs directly pretreated with TGF-β could promote EMT in pancreatic cancer cell lines [34]. TNF-α promotes the expression of the CXCR3 ligands CXCL9, CXCL10 and CXCL11 in MSCs and thereby promotes migration in breast cancer cell lines [82].

#### Inflammatory cytokines promote the secretion of angiogenic factors by MSCs

Pretreatment with IFN-γ and TNF-α induces VEGF expression in MSCs via the HIF-1 α signaling pathway, thereby enhancing the ability of MSCs to promote tumor angiogenesis. Inhibiting HIF-1α expression in MSCs abolishes the ability of MSCs to promote colon cancer growth in an inflammatory tumor microenvironment [75].

#### Treatment of MSCs with inflammatory cytokines promotes the expression of cancer stem cell-related genes

TGF-β-pretreated MSCs can increase the expression of various stemness-related proteins such as CD133, Nanog, and octamer-binding transcription factor 4 in in pancreatic cancer cells and enhance the sphere-forming capacity of these cancer cells [77].

#### The effects of tumor-secreted exosomes on MSCs

Exosomes are small vesicles (typical diameter, 30–100 nm) that are secreted by a variety of living cells. The exosomal lumen mainly contains cytoplasm; thus, exosomes can transport various molecules between cells. Exosomes derived from breast and ovarian cancer cells can cause adipose-derived MSCs to adopt TAF phenotypes, with upregulated α-SMA expression. Additionally, exosomes can promote expression of the tumor-associated factors such as SDF-1, VEGF, CCL5, and TGF-β in MSCs [78,83].

## Conclusion

Tumors cause inflammation, and inflammatory tumor microenvironments recruit MSCs from the circulation and adjacent tissues to tumor tissues, thereby educating these MSCs to adopt tumor growth-promoting phenotypes. MSCs in tumor tissues recruit additional immunosuppressive cells, resulting in the formation of an immunosuppressive microenvironment in the tumor vicinity. MSCs can also enhance the proportions of cancer stem cells in tumors, promote EMT, and stimulate tumor angiogenesis. These effects contribute to tumor growth, progression, and metastasis, leading to the formation of a vicious cycle.

However, given the plasticity of MSCs, alterations to the activated signaling pathways could potentially convert these MSCs to phenotypes that would inhibit tumor growth and development. Studies of not only the interactions among tumors, MSCs, and the inflammatory tumor microenvironment but also of methods to interrupt these interactions will likely provide new cancer therapy targets.

## Abbreviations

AMSC: Adipose derived mesenchymal stem cell; BAMB: Bone morphogenetic protein and activin membrane-bound inhibitor; CCL5: Chemokine (C-C motif) Ligand 5; CCR: C-C chemokine receptor; CXCR: chemokine (C-X-C motif) receptor; CXCL: c-x-c motif chenokine; DC: Dendritic cells; EC: Endothelial cell; EMT: Epithelial-mesenchymal transition; ERK: Extracellular signal-regulated kinase; ET-1: Endothelin-1; FAKs: Focal adhesion kinases; HIF-1: Hypoxia inducible factor 1; IDO: Indolamin2,3-dioxygenase; IL: Interleukin; IFN-γ: Interferon-γ; Jak2: Janus kinase 2; MAPK: Mitogen activated protein kinase; MDSC: Myeloid-derived suppressor cells; MMP: Matrix metalloproteinase; MSC: Mesenchymal stem cell; NK cells: Natural killer cells; NF-kB: Nuclear factor-kappa B; N-MSC: Native mesenchymal stem cell; PGE2: Prostaglandin E2; PGF: Placental growth factor; SDF-1: Stromal cell-derived factor-1-alpha; STAT3: Signal tranducers and transcription activators 3; SMA: Smooth muscle actin; TAF: Tumor related fibroblasts; TGF-β: Transforming growth factor-beta; TLR: Toll-like receptor; T-MSC: Tumor derived mesenchymal stem cell; TNF-α: Tumor necrosis factor-α; VCAM: Vasculaer cell adhersion molecule; VEGF: Vascular endothelial growth factor.

## Competing interests

The authors declare that they have no competing interests.

## Authors’ contributions

All authors contributed to the conception and design of this review, participated in the drafting of the manuscript, and approved its final version.
